# *Borrelia burgdorferi* Requires Glycerol for Maximum Fitness During The Tick Phase of the Enzootic Cycle

**DOI:** 10.1371/journal.ppat.1002102

**Published:** 2011-07-07

**Authors:** Christopher J. Pappas, Radha Iyer, Mary M. Petzke, Melissa J. Caimano, Justin D. Radolf, Ira Schwartz

**Affiliations:** 1 Department of Microbiology and Immunology, New York Medical College, Valhalla, New York, United States of America; 2 Department of Medicine, University of Connecticut Health Center, Farmington, Connecticut, United States of America; 3 Department of Genetics and Developmental Biology, University of Connecticut Health Center, Farmington, Connecticut, United States of America; 4 Department of Pediatrics, University of Connecticut Health Center, Farmington, Connecticut, United States of America; Medical College of Wisconsin, United States of America

## Abstract

*Borrelia burgdorferi*, the spirochetal agent of Lyme disease, is a vector-borne pathogen that cycles between a mammalian host and tick vector. This complex life cycle requires that the spirochete modulate its gene expression program to facilitate growth and maintenance in these diverse milieus. *B. burgdorferi* contains an operon that is predicted to encode proteins that would mediate the uptake and conversion of glycerol to dihydroxyacetone phosphate. Previous studies indicated that expression of the operon is elevated at 23°C and is repressed in the presence of the alternative sigma factor RpoS, suggesting that glycerol utilization may play an important role during the tick phase. This possibility was further explored in the current study by expression analysis and mutagenesis of *glpD*, a gene predicted to encode glycerol 3-phosphate dehydrogenase. Transcript levels for *glpD* were significantly lower in mouse joints relative to their levels in ticks. Expression of GlpD protein was repressed in an RpoS-dependent manner during growth of spirochetes within dialysis membrane chambers implanted in rat peritoneal cavities. In medium supplemented with glycerol as the principal carbohydrate, wild-type *B. burgdorferi* grew to a significantly higher cell density than *glpD* mutant spirochetes during growth in vitro at 25°C. *glpD* mutant spirochetes were fully infectious in mice by either needle or tick inoculation. In contrast, *glpD* mutants grew to significantly lower densities than wild-type *B. burgdorferi* in nymphal ticks and displayed a replication defect in feeding nymphs. The findings suggest that *B. burgdorferi* undergoes a switch in carbohydrate utilization during the mammal to tick transition. Further, the results demonstrate that the ability to utilize glycerol as a carbohydrate source for glycolysis during the tick phase of the infectious cycle is critical for maximal *B. burgdorferi* fitness.

## Author Summary

*Borrelia burgdorferi* is the vector-borne pathogen that causes Lyme disease. It has a complex life cycle that involves growth in a tick vector and a mammalian host — two diverse environments that present *B. burgdorferi* with alternative carbohydrate sources for support of growth. Previous studies suggested that glycerol may be an important nutrient in the tick vector. Here we show that genes predicted to be involved in glycerol metabolism have significantly elevated expression during all tick stages. Repression of expression in the mammalian host is dependent on the alternative sigma factor, RpoS. A mutant that cannot convert glycerol into dihydroxyacetone phosphate to support glycolysis was able to infect mice. In contrast, the mutant was present at significantly lower levels in nymphal ticks, its replication was delayed during nymphal feeding and longer feeding times were required for transmission from nymph to mouse. The results demonstrate that the ability to utilize glycerol as a carbohydrate source for glycolysis during the tick phase of the infectious cycle is critical for maximal *B. burgdorferi* fitness.

## Introduction

*Borrelia burgdorferi* is the spirochetal agent of Lyme disease, the most frequently reported vector-borne disease in the United States [1]. In the Northeastern United States, *B. burgdorferi* is transmitted between mammalian hosts by the bite of the black legged deer tick, *Ixodes scapularis*, with the white-footed mouse (*Peromyscus leucopus*) serving as the primary reservoir host [2], [3]. The transmission cycle is as intricate as the life of the tick itself. *B. burgdorferi* are acquired by uninfected larvae feeding on an infected small mammal [4]. This is essential for the continued maintenance of *B. burgdorferi* in nature, since there is no transovarial transmission in *Ixodes spp.* [5], [6]. The bacteria remain in the midgut of engorged larval ticks through the molt. The infected nymph will take a blood meal on a mammal, at which point *B. burgdorferi* multiply and begin their migration from the tick midgut to the salivary glands from which they are transmitted to a mammalian host [7]–[9], thereby completing the enzootic cycle.

*B. burgdorferi* must adjust its gene expression program in response to the different physiological cues encountered during the natural enzootic cycle. In bacteria, regulation of gene expression in response to environmental cues is often mediated by two-component systems (TCS) and/or alternative sigma factors [10], [11]. The *B. burgdorferi* genome encodes only two alternative sigma factors and two TCS [12], [13]. Thus, *B. burgdorferi* must orchestrate its complex expression programs with a limited repertoire of known transcriptional regulators. Studies by Norgard and co-workers demonstrated a link between one TCS, Hk2-Rrp2, and the alternative sigma factors RpoN and RpoS [14], [15]. The expression of several virulence genes, including *ospC*, *dbpA* and *bbk32*, are dependent on RpoS [14], [16], [17]. RpoS is also essential for repression of genes whose expression is required during the tick phase, but not in the mammalian host [17], [18]. BB0647 (BosR, Fur) has also been shown to play a role in RpoN-dependent expression of *rpoS* [19]–[21]. Less is currently known regarding the second TCS, consisting of Hk1 and Rrp1, but recent studies have begun to elucidate the processes that are regulated by this TCS [22]–[24]. In particular, Rrp1 has been shown to be responsible for production of bis-(3′-5′)-cyclic dimeric guanosine monophosphate (c-di-GMP) and mutagenesis of Rrp1 results in alteration of expression for a substantial number of genes, including those involved in uptake and dissimilation of glycerol [22], [23], [25].

Different carbohydrates are selectively available to *B. burgdorferi* during its enzootic cycle. Glucose is the primary carbohydrate constituent in mammalian blood [26], [27] and *B. burgdorferi* can use glucose to support growth [28]. Ticks rely on a high concentration of carbohydrates and other nutrients available in the blood meal for molting and successful oogenesis [29], [30]. During feeding, ticks create a peritrophic matrix above the epithelial cell layer, which serves both as a compartment to trap the blood meal and as a barrier to prevent invasion by microorganisms that accompany the blood meal. However, the peritrophic matrix is permeable to hexose sugars [29], [31]. Once hexoses permeate across the matrix, they are sequestered by midgut epithelial cells during larval feeding [29], [31]. Consequently, nutrients present in the blood meal are rapidly depleted during larval feeding and are likely non-existent in an unfed nymphal midgut. Therefore, spirochetes resident in the midgut must identify and utilize alternative carbohydrates until the unfed nymph takes its next blood meal.

**Figure 1:**
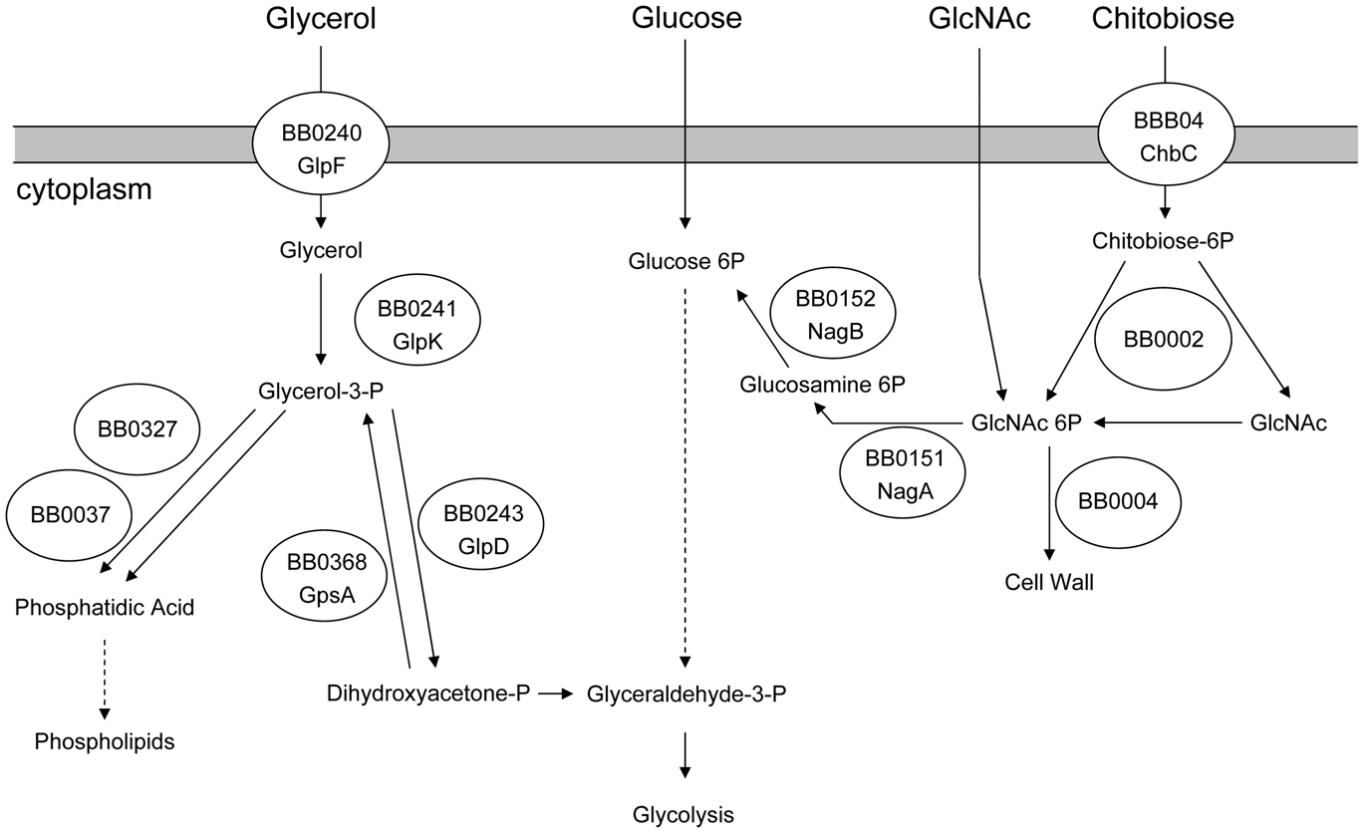
Predicted carbohydrate utilization pathways in *B. burgdorferi*. Based on Fraser et al. [12] and von Lackum and Stevenson [28].

Glycerol, a diffusible carbohydrate, is a readily available nutrient in the tick. Glycerol is produced by *Ixodes spp.* and serves as a colligative antifreeze for tick survival during the winter [32]–[34]. *B. burgdorferi* encodes a putative glycerol utilization operon consisting of three genes. *glpF* (*bb0240*) encodes a putative transmembrane facilitator protein that mediates the entry of free glycerol into the cell. *glpK* (*bb0241*) encodes a putative kinase that would produce glycerol 3-phosphate (G3P) which would be the substrate for glycerol 3-phosphate dehydrogenase (G3PDH), an enzyme putatively encoded by the third gene in the operon, *bb0243*. The resulting product, dihydroxyacetone phosphate, can enter glycolysis through the action of triose phosphate isomerase and ultimately result in the net production of one ATP molecule per original glycerol molecule [12], [28]. Alternatively, G3P may be converted to phosphatidic acid through the action of two enzymes, BB0327 (G3P acyltransferase) and BB0037 (Lysophosphatidic acid acyltransferase); this pathway is required for phospholipid biosynthesis and production of new cell membrane [12] (Figure 1).

Three lines of evidence suggest that glycerol utilization may be important during the vector phase of the enzootic cycle. Ojaimi et al. reported that all genes of the glycerol utilization operon are more highly expressed during in vitro growth in BSK-II medium at 23°C as compared to growth at 35°C [35]. Caimano et al. demonstrated that repression of *glp* operon expression is dependent on RpoS within the mammalian host [17]. Moreover, constitutive expression of the *glp* operon partially restores the ability of Rrp1-deficient *B. burgdorferi* to survive within feeding ticks [23].

In order to elucidate the role of glycerol uptake and utilization by *B. burgdorferi* during its natural life cycle and the regulatory events that govern *glp* operon expression, the gene predicted to encode G3PDH (*bb0243*) was disrupted and the effects of mutagenesis were evaluated in vitro and during infection of ticks or mice. The results demonstrate that the ability to utilize glycerol as a carbohydrate for use in the glycolytic pathway during the tick phase of the infectious cycle is critical for maximal *B. burgdorferi* fitness.

## Results

### Bioinformatic analysis of *B. burgdorferi* glycerol 3-phosphate dehydrogenase

The *B. burgdorferi* G3PDH genomic sequence has been annotated to putatively encode the anaerobic form of the enzyme based on its similarity to the anaerobic G3PDH ortholog of *Haemophilus influenzae* strain Rd (*glpA*) [12]. Other organisms containing an anaerobic GlpA (such as *E. coli*) contain two additional subunits as part of the functional G3PDH enzyme; GlpB, a subunit involved in FMN binding [36] and GlpC, a small membrane anchoring subunit [37]. Together, the individual protein molecules form a functional GlpABC heterotrimer [36]. Whole genome sequencing of *B. burgdorferi* failed to reveal putative genes with homology to any known *glpB* or *glpC* orthologs [12]. Further, BLASTP analysis revealed no orthologs in *B. burgdorferi* with similarity to either *E. coli* strain K12 or *H. influenzae* strain Rd GlpB or GlpC.

**Figure 2:**
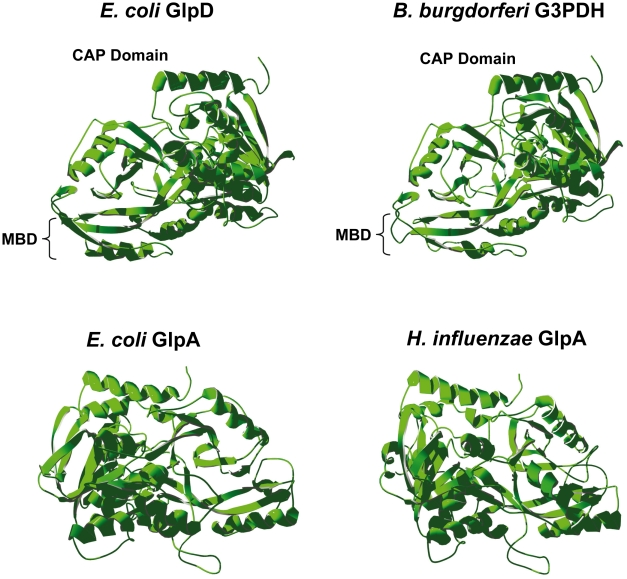
Predicted tertiary structure of *B. burgdorferi* G3PDH. The *B. burgdorferi* G3PDH sequence was mapped onto known bacterial G3PDH three-dimensional structures using SWISS-MODEL software. Structures were rotated so they are aligned in an identical orientation, with the C-terminal α-helix (red) at the top in all structures. MBD, putative membrane binding domain [41].

The tertiary structure of *B. burgdorferi* G3PDH was predicted using the SWISS-MODEL server [38]–[40] by comparison to *E. coli* K12 aerobic GlpD and anaerobic GlpA, as well as *H. influenzae* strain Rd anaerobic GlpA. *B. burgdorferi* G3PDH autoaligned with *E. coli* aerobic GlpD (PDB 2QCU), but not with *E. coli* GlpA [41] (Figure 2). *E. coli* anaerobic GlpA and *H. influenzae* GlpA auto-aligned to the anaerobic GlpA of *Bacillus halodurans* (PDB 3DA1) [42] (Figure 2). The modeling predicts that *B. burgdorferi* G3PDH and *E. coli* aerobic GlpD share similar tertiary structures. Yeh et al. have described 14 amino acid residues that participate in the *E. coli* GlpD active site based on a 1.75 Å structural model [41]. *B. burgdorferi* G3PDH contains conserved residues at 12/14 positions, in contrast to the *E. coli and H. influenzae* GlpA proteins (9/14). Taken together, the bioinformatic analyses suggest that *B. burgdorferi* G3PDH has greater similarity to aerobic forms of the enzyme. We propose that annotation of *bb0243* should be changed to indicate that it putatively encodes an aerobic GlpD and *B. burgdorferi* G3PDH is referred to as GlpD in the remainder of this report.

### *B. burgdorferi glpD* has elevated expression in ticks

**Figure 3:**
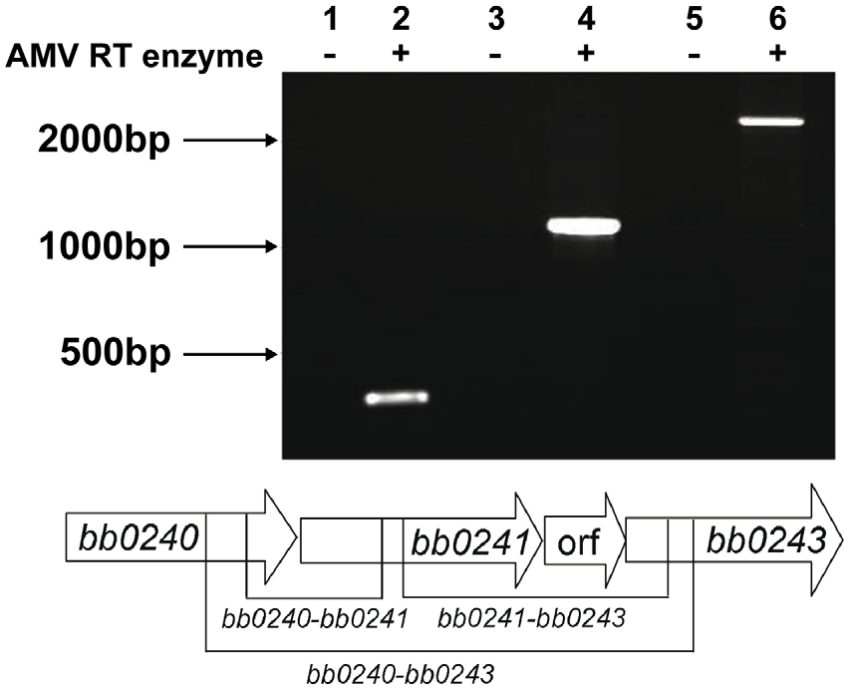
The *B. burgdorferi glp* region constitutes an operon. RT-PCR of RNA extracted from B31-A3 grown at 37°C in BSK-II medium was performed using primers listed in table 4. Lanes 1, 2: *bb0240*-*bb0241* (expected size 349 bp); lanes 3, 4: *bb0241*–*bb0243* (expected size 1141 bp); lanes 5, 6: *bb0240*–*bb0243* (expected size 2383 bp). Migration positions of DNA size markers are indicated on left. A schematic diagram showing the operon structure and sizes of expected amplification products is shown below the gel picture.

**Figure 4:**
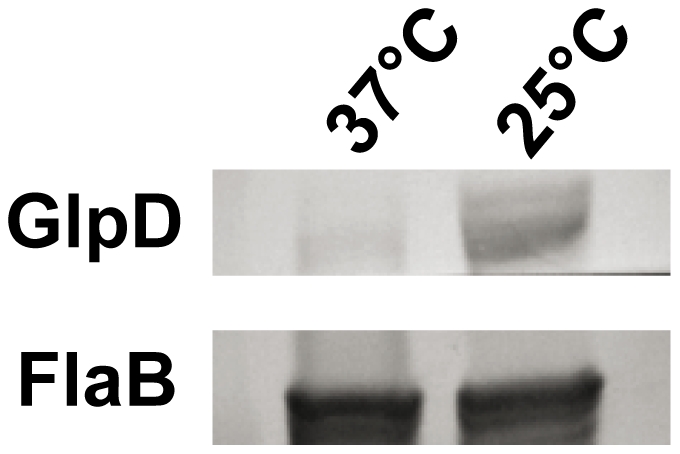
*B. burgdorferi* GlpD expression is temperature dependent. Whole cell lysates of *B. burgdorferi* B31-A3 grown at 37°C and 25°C were analyzed by immunoblotting. FlaB levels were determined as a control.

The physical linkage of *bb0240*, *bb0241*, and *bb0243* in the *B. burgdorferi* chromosome suggests that these genes comprise an operon [35]. RT-PCR analysis using RNA extracted from *B. burgdorferi* strain B31-A3 revealed that these genes are transcribed as a single operon (Figure 3). Ojaimi et al. reported that the *B. burgdorferi* glycerol operon is more highly transcribed at 23°C relative to transcript levels in cells grown at 35°C [35]. In order to explore if this increased transcript level is reflected in protein, strain B31-A3 whole cell lysate was tested to determine the protein expression levels at these two temperatures. Immunoblot analysis revealed that when *B. burgdorferi* strain B31-A3 was grown at 25°C, 7-fold more GlpD was generated compared to the level in cells grown at 37°C (Figure 4).

**Figure 5:**
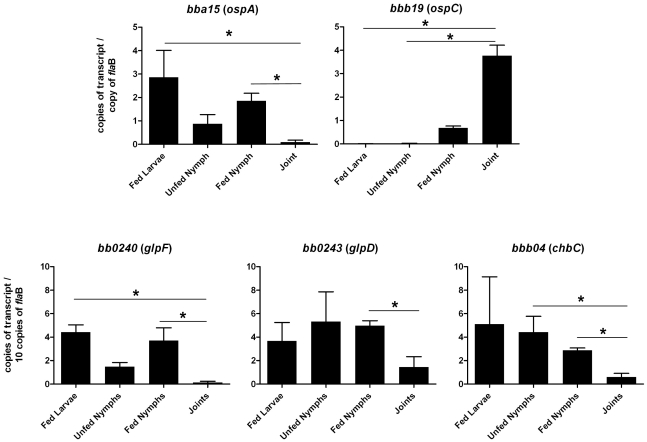
Transcriptional analysis of selected genes at different stages of the enzootic cycle. qRT-PCR was performed with RNA isolated from strain B31-A3-infected ticks and mouse joints as described in Methods. Statistical analysis was determined by Kruskal-Wallis multiple comparison Z- value test; * significant difference (*P*≤.05) between samples. Error bars indicate standard error of the mean (SEM).

**Figure 6:**
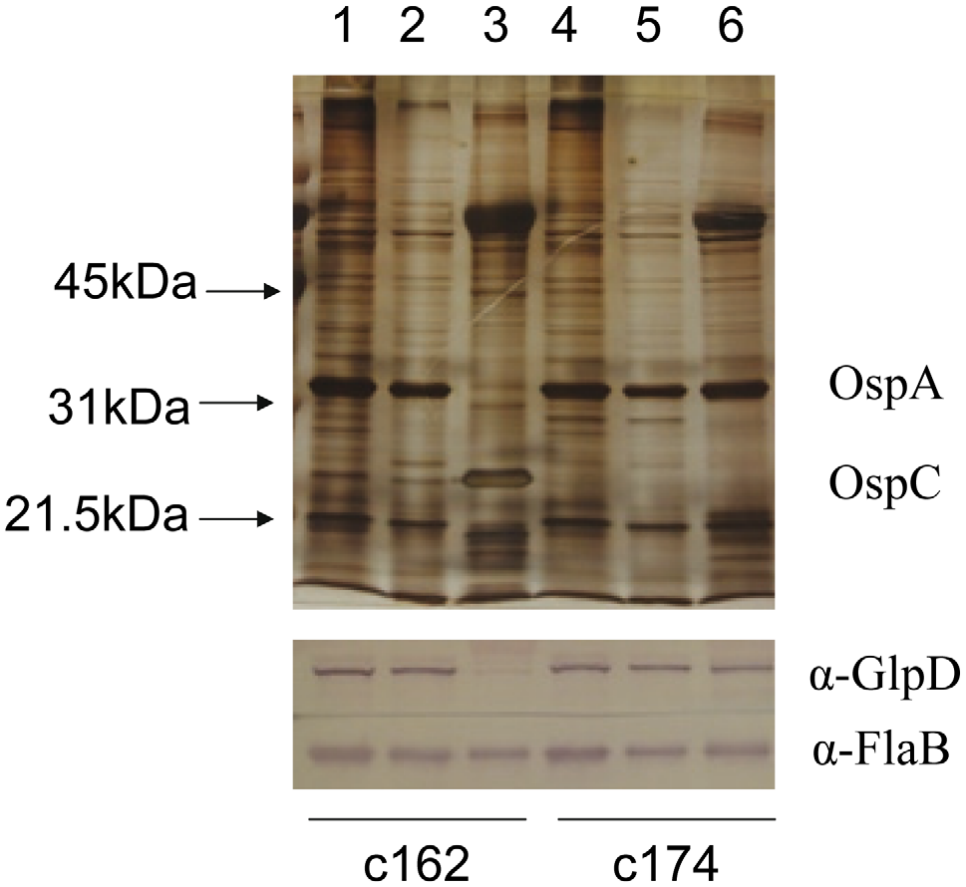
Repression of GlpD expression requires RpoS. Wild type (c162) and RpoS mutant (c174) *B. burgdorferi* were cultivated in vitro at either 23°C or 37°C or in DMC. Cell lysates were subjected to SDS-PAGE and gels were either silver stained (top panel) or blotted to PVDF membranes and developed with antibodies to GlpD or FlaB (bottom panel). Lanes 1 and 4, 23°C; lanes 2 and 5, 37°C; lanes 3 and 6, DMC. Migration positions of protein molecular mass markers are indicated on left in top panel.

To explore the possibility that expression may be differentially regulated in vivo, *glp* transcript levels were measured in infected ticks or mouse joints by real time RT-PCR; transcription of *ospA* and *ospC* was monitored as a control. Expression of the latter genes followed the expected pattern; *ospA* was expressed exclusively in ticks and *ospC* transcript was detected only in feeding nymphs and mouse joints (Figure 5). *glpD* expression was substantially higher during all tick stages (fed larvae, 3.68±2.72 copies/10 copies of *flaB*; unfed nymphs, 5.31±4.42; fed nymphs, 4.97±0.74) than in mouse joints (1.45±1.98 copies/10 copies of *flaB*). A similar expression pattern was observed for *glpF*, the first gene in the operon (fed larvae, 4.42±1.07 copies/10 copies of *flaB*; unfed nymphs, 1.48±0.61; fed nymphs, 3.70±1.89; mouse joint, 0.13±0.23) (Figure 5). Caimano et al. showed that transcription of *glp* operon genes is subject to RpoS-dependent repression [17]. This was confirmed at the protein level for GlpD as shown in Figure 6. Wild-type or RpoS mutant cells were grown in vitro at either 23°C or 37°C or in dialysis membrane chambers (DMCs) implanted in rat peritoneal cavities. Induction of OspC expression and repression of OspA expression in DMCs confirmed *that B. burgdorferi* attained the host-adapted state and abrogation of these changes in expression in the RpoS mutant showed that these alterations were dependent on RpoS, as expected. GlpD expression was virtually abolished in wild-type *B. burgdorferi* grown in DMC and this effect was not observed in the RpoS mutant cells (figure 6).

### Construction of a *B. burgdorferi glpD* mutant

**Figure 7:**
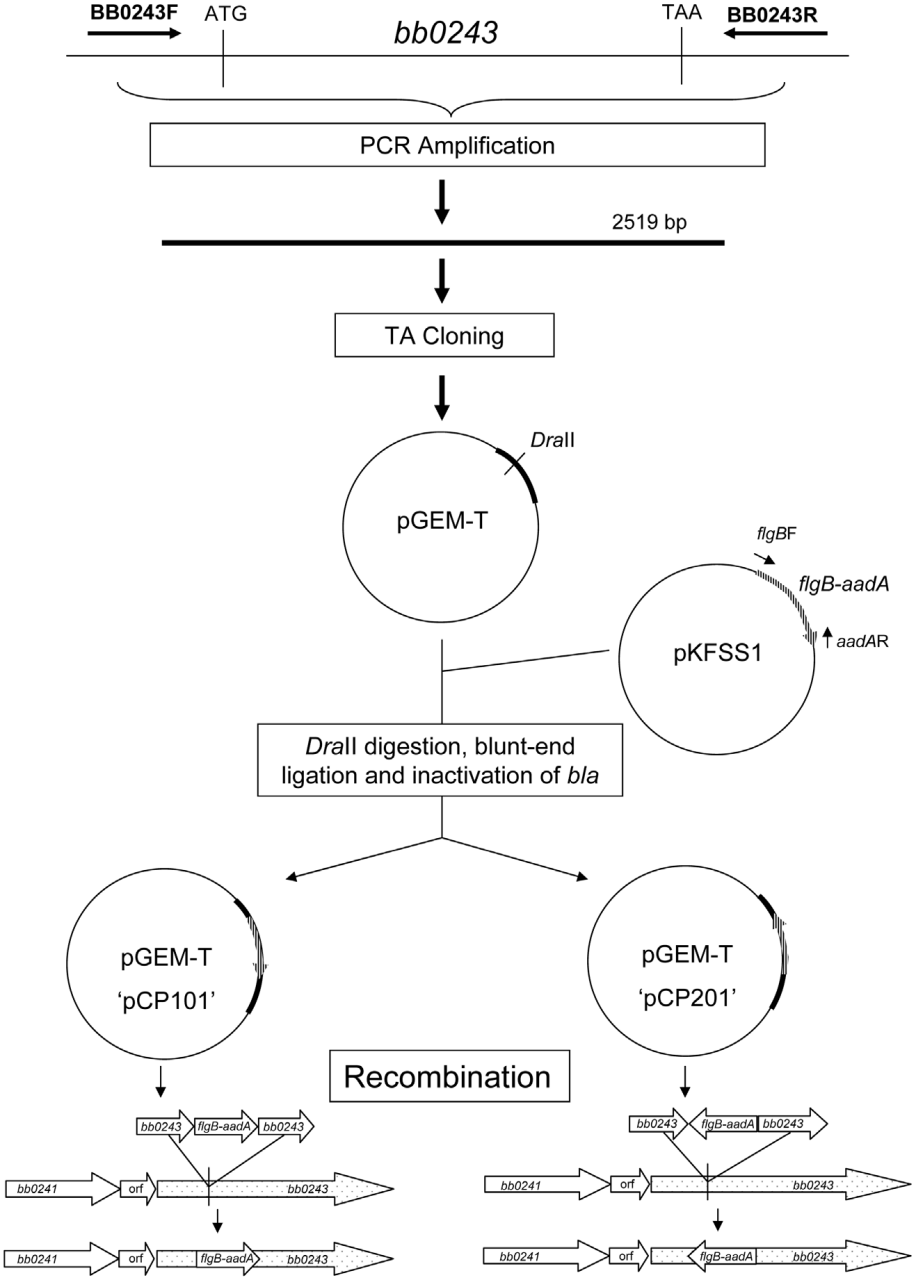
Strategy for generation of *glpD* disruption mutants. *glpD* mutant clones CP176 and CP177 were generated by recombination with pCP101; *glpD* mutant clone CP257 was generated by recombination with pCP201.

**Figure 8:**
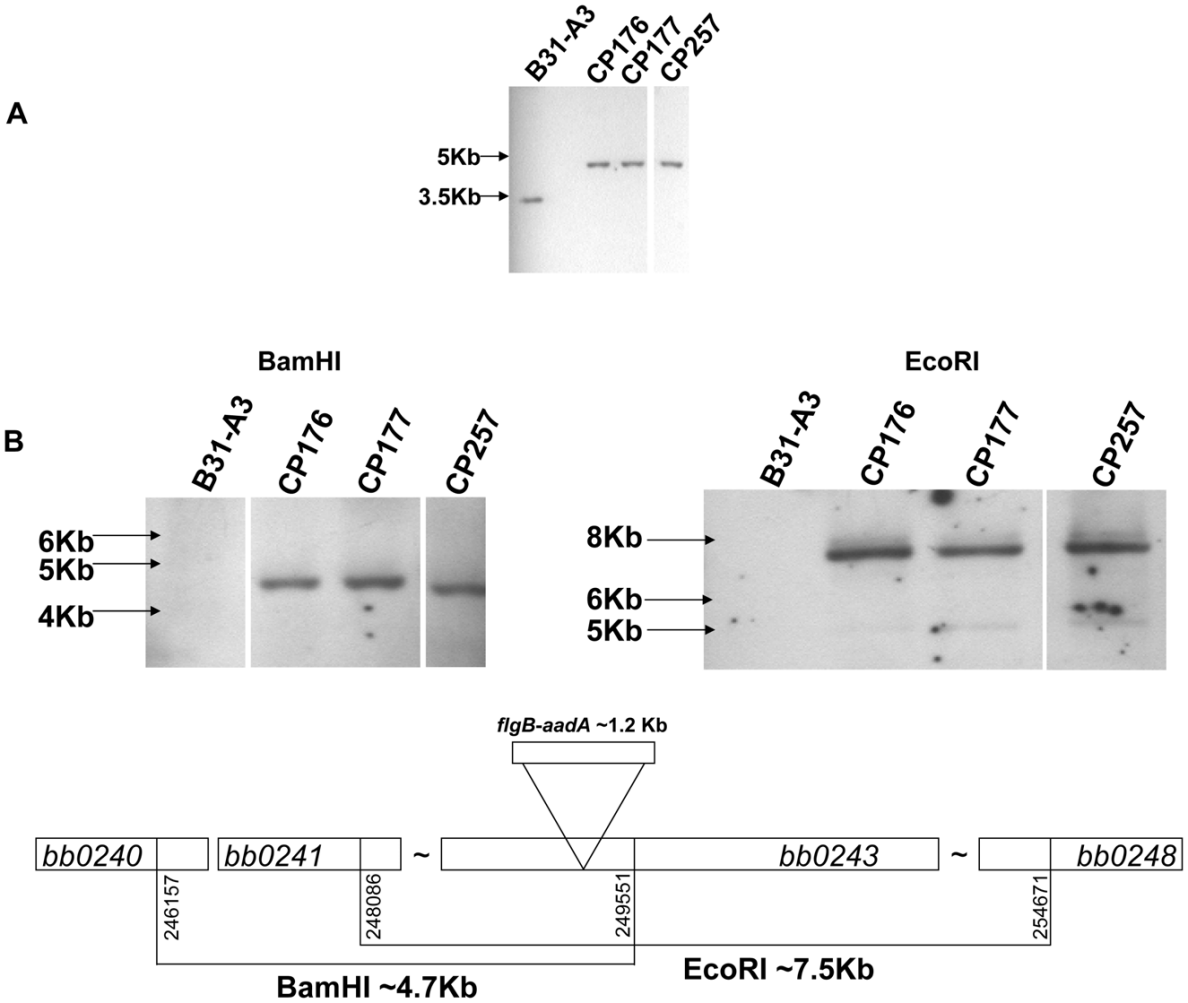
Confirmation of *glpD* disruption. **A**) Southern blot analysis confirms the disruption of *glpD*. Whole genomic DNA from B31-A3 and *glpD* disruption mutants CP176, CP177, CP257 were digested with BamHI and blotted on a nylon membrane. Digoxygenein-11-dUTP labeled *bb0243* probe was utilized to visualize *glpD*. Migration positions of DNA molecular size markers are indicated on the left. **B**) *glpD* disruption occurred via by a double crossover insertion of *flgB*-*aadA*. Whole genomic DNA from B31-A3 and *glpD* disruption mutants CP176, CP177, CP257 were digested with BamHI or EcoRI, which would be specific for the proximal or distal *bb0243* flanking chromosomal regions, respectively, and blotted to a nylon membrane. Blots were developed with a digoxygenein-11-dUTP-labeled *aadA* probe. A schematic diagram indicates the sizes of the fragments expected to contain *flgB*-*aadA*. Migration positions of DNA molecular size markers are indicated on the left of each panel.

**Figure 9:**
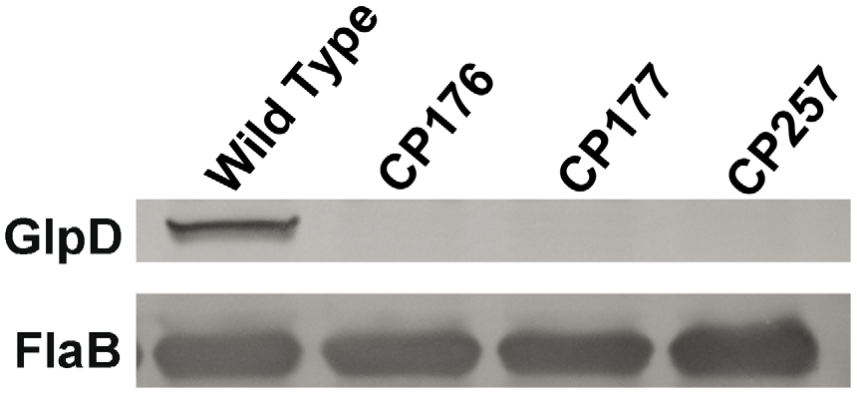
GlpD is absent in disruption mutants. Whole cell lysates from B31-A3, CP176, CP177, and CP257 were separated by SDS-PAGE, followed by immunoblot analysis using GlpD-specific antiserum. Presence of FlaB was also measured as a control.

To study the role of glycerol utilization in *B. burgdorferi*, *glpD*, the distal ORF in the glycerol operon was inactivated in strain B31-A3 by disruption with a *flgB-aadA* cassette inserted at residue K149 (Figure 7). Three mutants, two with the *flgB-aadA* cassette in the same orientation as the operon (CP176, CP177) and one with the insert in the opposite orientation (CP257), were isolated (Figure 8A). Southern blot analysis confirmed a disruption in *glpD* and showed that recombination occurred by a double crossover event in all three mutants (Figure 8B). Western blot analysis revealed that GlpD was absent in the mutants (Figure 9). Analysis of plasmid content of the wild type by PCR revealed that it lacked lp5 and cp9 and contained all other B31 linear plasmids, including those essential for murine infectivity. GlpD mutants had the same plasmid profile as the parental strain (data not shown).

Repeated attempts to isolate a complemented mutant strain were unsuccessful. Most experiments described below were carried out with all three isolated *glpD* mutants. Mutants CP176 and CP177 were isolated from one transformation and CP257 was obtained independently, thereby mitigating the concern that the observed mutant phenotypes were the result of a second site mutation.

### GlpD is required for maximal in vitro growth when glycerol is the principal carbohydrate source

To begin to characterize the role of GlpD in *B. burgdorferi* physiology, growth of *glpD* mutants was compared to that of wild-type B31-A3 in BSK-II, an undefined, enriched medium that contains glucose as the principal carbohydrate source [43]. No differences in final cell density were detected between wild-type and *glpD* mutants at either 25°C (1.3×10^9^ and 1.1×10^9^, respectively) or 37°C (1.4×10^9^ and 1.3×10^9^, respectively). In addition, no difference in growth characteristics was observed (data not shown).

**Figure 10:**
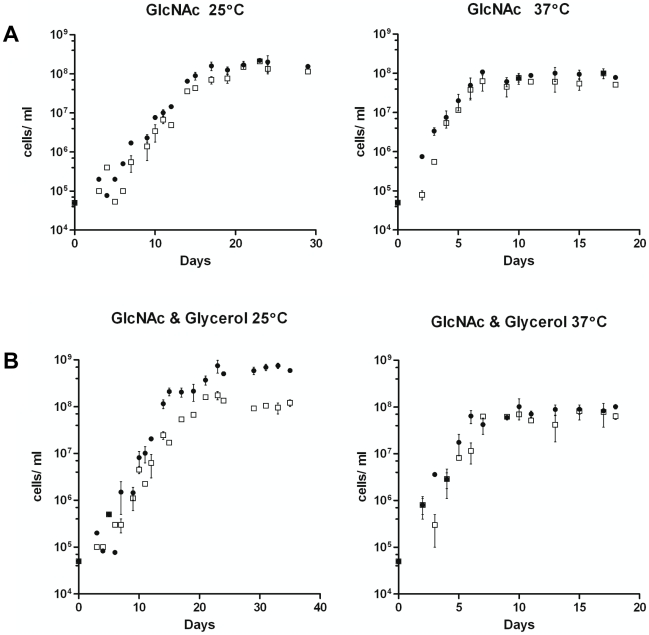
Growth of B31-A3 and CP176 in modified enriched medium. **A**) BSK-lite containing GlcNAc as the sole carbohydrate. •, B31-A3; □, CP176. Left panel, 25°C: B31-A3 achieved a stationary phase cell concentration of 1.8×10^8^ and CP176 achieved a stationary phase cell concentration of 1.5×10^8^. Right panel, 37°C: B31-A3 achieved a stationary phase cell concentration of 8.5×10^7^ and CP176 achieved a stationary phase cell concentration of 6.3×10^7^. All data points were determined in quadruplicate. Results are representative of two independent experiments. **B**) BSK-lite containing GlcNAc and glycerol as the principal carbohydrate. •, B31-A3; □, CP176. Left panel, 25°C: B31-A3 achieved a stationary phase cell concentration of 6.4×10^8^ and CP176 achieved a stationary phase cell concentration of 1.1×10^8^. The difference in stationary phase cell concentration between B31-A3 and CP176 at 25°C was significant (*P*<.001). Right panel, 37°C: B31-A3 achieved a stationary phase cell concentration of 9.1×10^7^ and CP176 achieved a stationary phase cell concentration of 6.4×10^7^. All data points were measured in quadruplicate. Results are representative of three independent experiments. Error bars indicate SEM.

Previous studies have shown that N-acetyl glucosamine (GlcNAc) is required for growth of *B. burgdorferi* in vitro [44]–[46]. There were no differences in growth characteristics between B31-A3 and CP176 grown in a modified BSK-II medium that did not contain glucose but had GlcNAc as the carbohydrate source (BSK-lite [28]) (Figure 10A). In contrast to growth in BSK-II with GlcNAc only or medium supplemented with glucose (data not shown), there was a significant difference in growth between wild type and *glpD* mutants when glycerol was supplied as the principal carbohydrate source. B31-A3 reached a significantly higher cell density in BSK-glycerol medium compared to CP176 when grown at 25°C (6.4×10^8^ and 1.1×10^8^, respectively; *P*<0.001) (Figure 10B). Interestingly, this effect was observed only at 25°C; when cultivated at 37°C, the growth characteristics of wild type and CP176 were indistinguishable. Indeed, B31-A3 cultures achieved significantly higher cell densities at 25°C as compared to 37°C in BSK-glycerol medium (6.4×10^8^ and 9.1×10^7^, respectively; *P*<0.001). These experiments were repeated with the other two independent *glpD* mutants (CP177, CP257) with essentially identical results (data not shown). These findings suggest that *B. burgdorferi* can utilize glycerol to support enhanced growth at the lower temperature. This observation would be consistent with the elevated expression of GlpD at 25°C (Figure 4).

### GlpD is not required for murine infection by *B. burgdorferi*

In order to determine whether the absence of GlpD affects the pathogenic properties of *B. burgdorferi*, C3H/HeJ mice were needle inoculated with 1×10^4^ cells of either wild type or *glpD* mutant. All mice in both the wild type and *glpD* mutant groups were infected and were seropositive by four weeks post-inoculation (Table 1). Unfed larvae were allowed to feed to repletion on these infected mice to allow spirochete acquisition by ticks. Infected fed larvae that molted to nymphs were fed to repletion on naïve C3H/HeJ mice. Viable spirochetes were recovered from all mice that were fed on by either wild type- or *glpD* mutant-infected ticks and seroconverted by 4 weeks post-feeding (Table 1). These results demonstrate that GlpD is not required for murine infection by *B. burgdorferi*. Further, GlpD-deficient spirochetes were acquired by larvae fed on infected mice, persisted through the molt and were transmitted to naïve mice by infected nymphs.

**Table 1:** Infectivity of *B. burgdorferi* wild type and GlpD mutants in mice.

Condition	Strain	Ear	Bladder	Joint
Needle Inoculated Mice^a^	Wild Type (B31-A3)	3/3	3/3	3/3
	CP176 (B31-A3Δ*glpD*)	3/3	1/3	3/3
	CP177 (B31-A3Δ*glpD*)	4/4	4/4	4/4
	CP257 (B31-A3Δ*glpD*)	3/3	1/3	3/3
Nymph Infected Mice^b^	Wild Type (B31-A3)	3/3	3/3	3/3
	CP176 (B31-A3Δ*glpD*)	4/4	4/4	4/4
	CP257 (B31-A3Δ*glpD*)	3/3	3/3	3/3

Table represents typical results from 1 of 3 independent experiments.

a Wild-type strain B31-A3 or GlpD mutants were needle inoculated into naïve C3H/HeJ mice and tested for infectivity by cultivation of indicated tissue in BSK medium.

b Unfed nymphs naturally infected with either wild-type or GlpD mutant strains were placed on naïve C3H/HeJ mice.

### *glpD* mutants have a lower spirochete density in unfed nymphs

The enhanced growth of *B. burgdorferi* at lower ambient temperature in vitro when glycerol is the principal carbohydrate source, elevated expression of GlpD at the lower temperature and its RpoS-dependent repression in DMCs suggested that glycerol may be an important nutrient for *B. burgdorferi* during the tick phase of its life cycle. Therefore, the effect of *glpD* disruption was explored more extensively in infected ticks. Naïve, unfed larvae were placed on mice infected with either B31-A3 or CP176, allowed to feed until repletion and molt to the nymphal stage. Spirochete loads in infected ticks were measured by qPCR (Table 2). Larvae infected with either B31-A3 or CP176 had similar spirochete loads (approximately 700 spirochetes/larvae).

**Table 2:** Spirochete density at different tick phases.

Condition	Strain	Spirochetes/Tick	SD	*P* value^a^
Fed Larvae	Wild Type (B31-A3)	737	±369	0.5646
	CP176 (B31-A3 Δ*glpD*)	632	±343	
Unfed Nymphs	Wild Type (B31-A3)	1173	±637	2.76E-08
	CP176 (B31-A3Δ*glpD*)	254	±137	
Engorged Nymphs	Wild Type (B31-A3)	56825	±47640	0.0198
	CP176 (B31-A3Δ*glpD*)	17138	±22000	

Spirochete density was measured by qPCR.

a Student's *t*-test comparing wild type to CP176.

Spirochete numbers in wild type-infected ticks increased slightly after larval molting to nymphs but decreased in nymphs infected with any of the *glpD* mutant strains. This resulted in a significant five-fold decrease in CP176 density in unfed nymphs compared to the wild-type (Table 2). Further, in independent experiments, CP177 and CP257 had an identical phenotype to that observed for CP176, i.e. spirochete densities were significantly lower after molting as compared to spirochete loads in B31-A3-infected ticks (data not shown).

Infected nymphs were fed on naïve mice and spirochete loads were measured in the resulting fed nymphs. As expected, spirochete numbers increased substantially during nymphal feeding in both wild type- and CP176-infected nymphs, although the spirochete burdens in the *glpD*-infected engorged nymphs were significantly lower than in nymphs infected with the parental strain (*P*<.02) (Table 2). These results suggest a role for glycerol utilization by *B. burgdorferi* as an important factor for spirochete maintenance during transtadial transition.

### Absence of GlpD results in impaired *B. burgdorferi* replication in feeding nymphs

As previously described, wild type- and *glpD* mutant-infected nymphs are equally capable of transmitting *B. burgdorferi* to, and causing infection in, mice (Table 1). However, those studies were conducted by allowing nymphs to feed to repletion. A feeding nymph will attach and feed on a host for 72 hours or longer [47]. During this time, replicating *B. burgdorferi* surround midgut epithelial cells, penetrate the midgut basement membrane, and enter the hemocoel and salivary glands from which they are ultimately transmitted to the mammal [8], [9]. Therefore, replication is a critical step in spirochete transmission from the vector to the mammalian host. Since the density of *glpD* mutant spirochetes decreases as a result of molting and was five-fold lower than in wild type-infected unfed nymphs, we reasoned that nymphs harboring *glpD* mutant spirochetes would require a longer feeding period before transmission to the host due to its delayed exit from the tick midgut.

**Figure 11:**
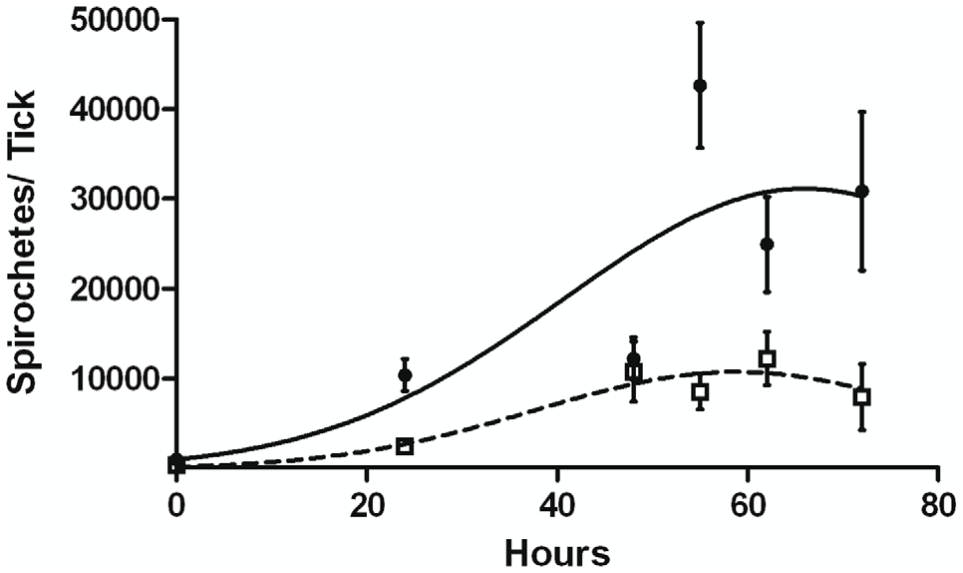
Replication of B31-A3 or CP176 in feeding nymphs. B31-A3- or CP176- infected nymphs were placed on naïve mice, removed at the indicated times and spirochete density was determined by qPCR. •, B31-A3; □, CP176. Spirochete densities for B31-A3 and CP176 were significantly different from each other (*P*<.05) at all time points except 48 hours. Error bars indicate SEM.

To explore this possibility, B31-A3- and CP176-infected nymphs were placed on naïve mice, allowed to begin feeding, but forcibly removed at different time points post-attachment. Mice were then monitored for evidence of infection. In a pilot experiment, nymphs were fed on naïve mice for 65 hours; 2/2 mice fed on by B31-A3-infected nymphs became infected, whereas 0/3 mice fed on by CP176-infected nymphs acquired infection. Based on this pilot study, B31-A3- or CP176-infected unfed nymphs were placed on the outer ear of naïve C3H/HeJ mice and allowed to feed for either 24, 48, 55, 62 or 72 hours or collected at drop off (>72 hours). Ticks were removed at each time point and spirochete load was determined by qPCR. Results presented in Figure 11 demonstrate that CP176 experienced a lag in replication and achieved significantly lower spirochete loads at times beyond 48 hours of feeding (*P*<0.001). At these points, spirochete loads per tick were 3.5–5 fold lower in CP176-infected ticks than in those infected with B31-A3 (e.g., at 55 hours of feeding spirochete loads were 42,633 and 8,581 for B31-A3 and CP176-infected nymphs, respectively) (Figure 11). Mice were also monitored for infection. Results demonstrate that mice fed on by wild type-infected nymphs were infected by 62 hours of feeding. In contrast, CP176-infected nymphs produced infection in mice only after at least 72 hours of feeding (Table 3). These data suggest that wild type-infected nymphs are more readily able to infect naïve mice due to a more rapid increase in spirochete density induced on commencement of tick feeding. Further, disruption of glycerol utilization results in reduced fitness of the spirochete during the tick phase and spirochetes that are unable to utilize glycerol are at a disadvantage for transmission to a mammalian host.

**Table 3:** Kinetics of Mouse Infectivity by Tick Feeding.

Time Attached	48 hours	55 hours	62 hours	72+ hours
**Wild Type**	0/2	0/1^a^	3/3	2/2
**CP176**	0/2	0/3	0/3	2/2

a Two mice died.

### Expression of *chbC*, encoding a chitobiose transporter subunit, is elevated in ticks

Chitobiose is a di-GlcNAc molecule that is a component of the peritrophic matrix and tick chitin [45], [46], [48], [49]. The *B. burgdorferi* genome contains open reading frames that encode gene products that can mediate chitobiose transport and metabolism [12], [46], [48], [49]. These include the three subunits of a chitobiose transporter (BBB04-BBB06), a putative chitobiase (BB0002) and NagA and NagB (BB0151 and BB0152). In combination, the actions of these gene products would result in production of glucose 6-phosphate that could enter the glycolytic pathway (Figure 1). Both chitobiose and chitin can support *B. burgdorferi* growth in vitro [45], [46], [48], [49]. Therefore, the transcript levels for *chb*C *(bbb04)*, which encodes subunit C of the chitobiose transporter, were also measured to determine whether chitobiose utilization by *B. burgdorferi* may be important during the tick phase of the enzootic cycle. Substantially higher expression of *chb*C occurred during the various tick stages (fed larvae, 5.10±7.00 copies/10 copies of *flaB*; unfed nymphs, 4.42±2.33; fed nymphs, 2.87±0.36) as compared to expression in mouse joints (0.59±0.71 copies/10 copies of *flaB*) (figure 5).

## Discussion

On acquisition by feeding larvae from an infected mammal, *B. burgdorferi* must initially adapt to the new host (i.e. tick) environment. The spirochete must then survive the tick molting process and endure a substantial period in a nutrient-poor milieu (unfed nymph). This is not a period of metabolic dormancy since several studies, including our own, demonstrate that *B. burgdorferi* gene expression is modulated in different tick developmental stages and that expression of some genes is higher in unfed nymphs than in fed nymphs [17], [50], [51]. During the subsequent nymphal blood meal, *B. burgdorferi* enter a rapid replication phase, experiencing a significant increase in density within a 48 hr period [8], [9] and must prepare for transmission back to a mammal. How does *B. burgdorferi* generate the energy required to withstand this harsh environment?

Glycerol and its metabolites play important roles in cellular biochemistry [52] and glycerol is a readily available carbohydrate in *Ixodes* ticks [32]–[34]. Most bacteria have the ability to acquire glycerol from the surrounding milieu or to re-utilize it from its own metabolites [52], [53]. G3P is a crucial intermediate for energy metabolism (via its conversion to dihydoxyacetone phosphate and entry into the glycolytic pathway) and for phospholipid biosynthesis (via its conversion to phosphatidic acid [Figure 1]). The *B. burgdorferi* genome putatively encodes all the enzymes required for both processes [12], [54]. The possibility that glycerol uptake and utilization may play an important role during the tick phase of the enzootic cycle was suggested by previous studies showing that genes comprising the *glp* operon had elevated expression during growth in vitro at 23°C and were subject to RpoS-dependent repression within the mammalian host [17], [35]. A number of findings from the current study confirm that this is the case. First, in medium supplemented with glycerol as the principal carbohydrate source, wild-type *B. burgdorferi* grew to a significantly higher cell density compared to a *glpD* mutant during growth at 25°C (Figure 10B). This difference was not observed during growth at 37°C or when glucose was employed as the principal carbohydrate source. Second, transcript levels for *glpF* and *glpD* were significantly lower in mouse joints relative to their levels in ticks (Figure 5). Third, GlpD protein was not produced during growth in DMCs and its repression was dependent on the presence of RpoS (Figure 6). Finally, the *glpD* mutant was fully infectious in mice when introduced by either needle or tick inoculation (Table 1), but had a replication defect in ticks (Table 3 and Figure 11).

Absence of GlpD results in reduced spirochete fitness in the tick. This defect is manifested at two distinct points during this phase of the enzootic cycle. Spirochete loads are reduced five-fold after the larval molt in the mutant relative to the wild type. Spirochete loads were measured in larvae that had fed to repletion and in unfed nymphs two weeks after the molt. Therefore, it is not clear whether the reduction in *B. burgdorferi* density occurred during the molt or during the initial period in the unfed nymph. We favor the latter possibility. At the onset of nymphal feeding, the *glpD* mutants display a lag prior to beginning replication; as a result, they replicate more slowly than wild-type spirochetes and fail to achieve the same final spirochete densities (Figure 11). As a consequence, there is delayed transmission of *glpD* mutant spirochetes to mice during feeding. Whereas ticks infected with wild-type *B. burgdorferi* caused infection by 62 hours of feeding, those infected with mutant spirochetes required at least 72 hours of feeding before productive transmission occurred. Dunham-Ems et al. have demonstrated that *B. burgdorferi* migration from the midgut to the salivary glands for transmission to a mammal proceeds in two phases. In the initial step, replicating spirochetes form non-motile networks that advance toward the basolateral surface of the gut epithelium. The non-motile spirochetes then transition to motile organisms that penetrate the basement membrane into the hemocoel and migrate to the salivary gland [9]. This model of *B. burgdorferi* dissemination provides an explanation for the delayed transmission phenotype of the *glpD* mutant. As dissemination of *B. burgdorferi* in the tick during the first phase of feeding does not depend on motility, but instead is replication driven, the reduced replication rate of the mutant would result in delayed dissemination to the hemocoel. As a result, the mutant would require additional time for successful tick to mammal transmission.

Spirochete loads of wild type and *glpD* mutant were identical in fed larvae whereas those of the mutant were reduced approximately five-fold after the larval molt; the reduced level of mutant persisted throughout the subsequent stages of the tick cycle (Table 2). In a very recent study, He et al. showed that a *glpF* polar deletion mutant, which does not express any of the *glp* operon genes, had a phenotype both in vitro and in vivo very similar to that described here for a *glpD* mutant (i.e. the mutant failed to reach the same cell density as the wild type when cultured in medium with glycerol as the principal carbohydrate and had reduced spirochete loads in infected nymphs) [23]. Interestingly, the reduction in mutant spirochete levels in nymphs was much more severe (>2 logs) in their study than was observed here for the *glpD* mutant. Presumably, this difference is due to the fact that the *glpD* mutant will only have an effect on glycerol utilization for glycolysis, whereas the *glp* operon mutant will also affect phospholipid biosynthesis (Figure 1). Thus, study of the *glpD* mutant is important in allowing evaluation of the contribution of glycerol utilization for energy metabolism without any confounding from effects on other metabolic pathways.

Why isn't the *glpD* mutation lethal rather than being simply growth inhibitory? Clearly, there must be an alternative carbohydrate source that can be metabolized via glycolysis to produce the required ATP. We propose that this alternative energy source is chitobiose. Tilly et al. reported that *chbC* transcript is elevated at 23°C relative to 34°C [45] and we have found that *chbC* expression is significantly higher in ticks than in mouse joints (Figure 5). Taken together, these data suggest that chitobiose utilization by *B. burgdorferi* is important during the tick phase of the cycle. Chitobiose would be available to the spirochete during tick feeding, when it is shed from the forming peritrophic matrix, as well as during molting when the tick cuticle is being re-modeled for growth [31], [49]. It has been suggested that chitobiose utilization would be essential for *B. burgdorferi* during the tick phase of the enzootic cycle for spirochete glycolysis and cell wall synthesis. However, *chb*C mutants, which cannot take up exogenous chitobiose or utilize chitin to support growth, successfully complete the mouse-tick-mouse infectious cycle [48]. It is possible that glycerol availability may be partially responsible for rescue of the *chbC* mutant. This would suggest that *B. burgdorferi* maintains spirochete fitness in the nutrient deplete environment of the tick midgut by utilizing either glycerol and/or chitobiose as glycolytic precursors. It would be of interest to determine whether a *glpD-chbC* double mutant would be capable of completing the natural infectious cycle.

As described earlier, signals that lead to phosphorylation of Rrp2 result in activation of RpoN which, in turn, initiates transcription of *rpoS* [14], [15]. RpoS is expressed only in feeding nymphs and mammals and therefore, is thought to be responsible for the regulon that is required for mammalian infection [17], [55]. This is consistent with the fact that Rrp2, RpoN and RpoS mutants cannot establish infection in mice [16], [56], [57]. The RpoS regulon includes genes that are absolutely dependent on RpoS for their transcription (e.g. *ospC*), as well as genes subject to RpoS-dependent repression [17], [18]. The *glp* operon is in the latter category. Several recent studies have begun to reveal the role of the Hk1/Rrp1 TCS in *B. burgdorferi* [22]–[24]. Lack of Hk1 and Rrp1 has no effect on infectivity in mice. However, Hk1 and Rrp1 mutants are killed within the tick midgut during feeding [23], [24]. Hk1 and Rrp1 appear to be expressed during all stages of the *B. burgdorferi* life cycle [22], [24], but the important consideration is whether Rrp1 is phosphorylated leading to the production of c-di-GMP. Interestingly, Caimano et al. have recently shown that Hk1 mutants are killed during the larval and nymphal blood meals, indicating that c-di-GMP is required during both tick feeding stages [24]. It is reasonable to conclude that the Rrp2/RpoN/RpoS pathway governs the expression of *B. burgdorferi* genes required in the mammalian host, whereas Hk1/Rrp1 controls a subset of borrelial gene products that is critical for survival in the tick vector. A number of genes that are induced by Rrp1 (i.e. c-di-GMP) are subject to RpoS-dependent repression; these include the *glp* operon genes and *bba74* [22], [23].

**Figure 12:**
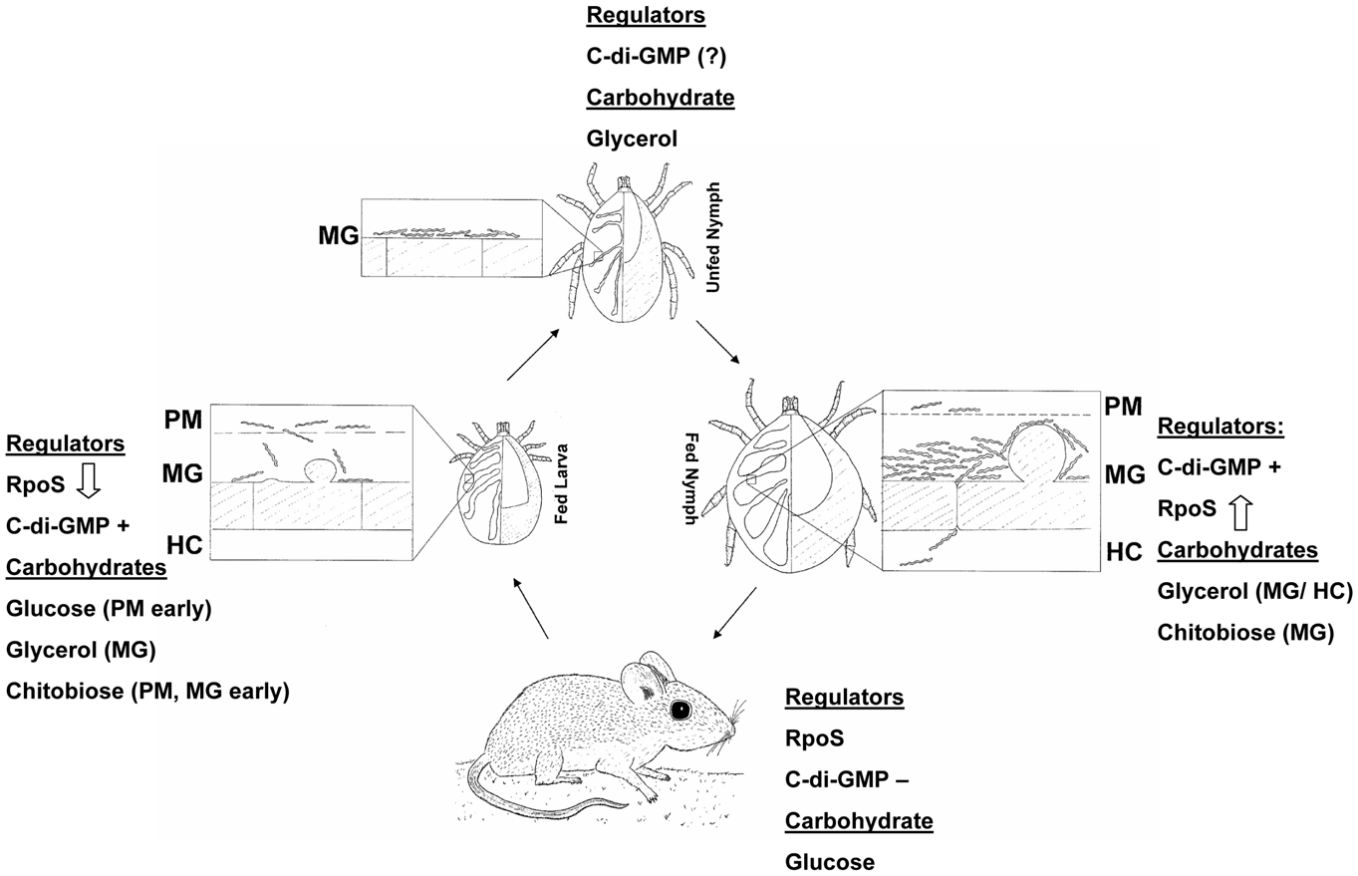
Carbohydrate availability and transcriptional regulators at different stages of the *B. burgdorferi* enzootic cycle. Up and down arrows indicate increasing or decreasing RpoS levels; c-di-GMP+ and c-di-GMP− indicate presence or absence of c-di-GMP. PM, peritrophic matrix; MG, midgut; HC, hemocoel.

The current study demonstrates that *glp* operon expression is modulated by nutrient availability. Several reports have established that this operon is regulated in a reciprocal manner by RpoS and Rrp1 [17], [22], [23]. The *glp* genes are the first borrelial gene products linked to spirochete metabolism whose expression is subject to regulation by both *B. burgdorferi* TCSs. As such, these genes represent a valuable paradigm for elucidating the interplay between these two regulatory pathways. A model that integrates both carbohydrate availability and presence/absence of transcriptional regulators is presented in Figure 12. It is presumed that early in larval feeding RpoS is still present, but must be degraded in order to allow for expression of tick phase genes; the precise timing of RpoS disappearance is not currently known. Glucose should be available at this stage in quantities sufficient to support *B. burgdorferi* growth. Later in the blood meal hexose sugars and other serum constituents crossing the peritrophic matrix are sequestered by tick midgut epithelial cells. This creates a nutrient-poor environment in which *B. burgdorferi* must rely on glycerol and rapidly depleting chitobiose to support glycolysis. The turnover of RpoS during larval feeding would result in the de-repression of glycerol pathway enzymes and presence of c-di-GMP will activate their expression, ensuring that spirochetes can rapidly switch from a glucose-based to a glycerol-based metabolism. Once spirochete infection is established in the midgut and the larva molts to an unfed nymph, *B. burgdorferi* remains a metabolically active spirochete that must rely on glycolysis for maintenance of cellular integrity. Glycerol is presumed to be the primary carbohydrate at this stage and absence of RpoS would allow expression of the *glp* operon. Early in nymphal feeding while blood constituents are scarce, *B. burgdorferi* must actively replicate and migrate through the epithelial midgut lumen to begin its migration to the salivary glands. At this point glycerol would be a primary energy source for support of cellular replication, as chitobiose will not be available until the eventual breakdown of the peritrophic matrix. Inability to utilize glycerol, as in the *glpD* mutant, would result in delayed and reduced spirochete replication that could impact transmission of *B. burgdorferi* to the mammalian host. Presence of c-di-GMP would result in sustained expression of *glp* operon genes. As RpoS levels increase during the nymphal blood meal, presence of c-di-GMP will counteract the repressive effects of RpoS on *glp* gene expression, ensuring that glycerol utilization can continue until the spirochetes are transmitted to a mammalian host.

The model presented in Figure 12 accounts for carbohydrate source availability and presence of regulatory molecules throughout the tick-mouse enzootic cycle and highlights the interdependence of these two parameters. Concentrations of glucose and glycerol in mouse plasma are approximately 150 and 2.8 mg/100 mL, respectively [58]. Glycerol is abundantly present during all tick stages. On this basis, the model assumes that *B. burgdorferi* utilizes glucose as the preferred nutrient source when it is available in the mammal or at certain stages during the tick blood meal, but switches to utilizing glycerol, especially in the unfed nymph when glucose is not present. This may represent the *B. burgdorferi* version of carbon catabolite repression (CCR), which is defined as a regulatory mechanism by which the expression and enzymatic activities of enzymes involved in the use of secondary carbohydrates are reduced in the presence of sufficient levels of the preferred carbohydrate [59]. The mechanisms underlying CCR in most bacteria involve a glucose-specific phosphotranferase subunit (EIIA) that can be reversibly phosphorylated based on the phosphoenolpyruvate to pyruvate ratios. *B. burgdorferi* encodes a putative EIIA subunit (BB0559), but does not appear to contain other major components that modulate CCR in other bacteria [12]. It is reasonable to assume that *B. burgdorferi* possesses sensing mechanisms that monitor the relative levels of glucose and glycerol in the environment. It is tempting to speculate that modulation of Hk1 kinase activity is one outcome of the fluctuating nutrient ratios. When the glycerol/glucose ratio increases Hk1 would phosphorylate Rrp1 leading to the production of c-di-GMP. The specific molecular signal that is recognized by Hk1 is not currently known. Likewise, the precise timing of the transcriptional activation/repression of RpoS and the possible reciprocal modulation of c-di-GMP levels is not known. Studies designed to elucidate the molecular events underlying the proposed model are warranted.

## Materials and Methods

### Ethics statement

All animal experimentation was conducted in strict accordance with the Guide for the Care and Use of Laboratory Animals of the National Institutes of Health. The protocols were approved by the Institutional Animal Care and Use Committee of New York Medical College (Approval number 31-1-0310H).

### Strains and growth conditions

*B. burgdorferi* strains B31-A3 [60], 297 (c162) and a strain 297-based RpoS mutant (c174) [17] were employed in this study. Spirochetes were grown in modified Barbour-Stoenner-Kelley-II medium [43], [61] supplemented with 6% heat inactivated rabbit serum (Sigma, St. Louis, MO) (BSK-II). BSK-lite medium was based on the formulation of Barbour [43] with modifications as previously described [28].

*B. burgdorferi* were grown to late log phase (5–10×10^7^ cells/ml) in BSK-II medium at 25°C. For BSK-lite experiments, spirochetes were diluted 100 fold in BSK-lite medium to remove BSK-II medium constituents. 5×10^4^ spirochetes in 40 ml of BSK-lite medium with a specific carbohydrate (either glucose or glycerol) were aliquoted into eight 5 ml tubes. Four tubes of each sample were placed at either 25°C or 37°C and observed for up to 60 days. Individual tubes were counted daily for cultures grown at 37°C and every two days for those incubated at 25°C. Spirochete density was enumerated by dark field microscopy as previously described [62]. Student's two-tailed, unpaired *t*-tests were performed on data collected during exponential phase and stationary phases of cell growth. Significance was defined as a *P*<0.01.

Cultivation of c162 and c174 in DMCs was carried out as described [63].

### Construction of a *B. burgdorferi glpD* mutant

The strategy for disruption of *B. burgdorferi* is presented in figure 7. A 2519 bp region of *B. burgdorferi* chromosomal DNA containing *bb0243* was amplified by PCR using primers bb0243F/R (Table 4) ligated into the pGEM-T vector (Promega, Madison, WI), transformed into *E. coli* DH5α and cells containing recombinant plasmids were selected by blue-white screening. Selected transformants were purified to single colonies and plasmids were confirmed to contain *bb0243* by DNA sequence analysis (Davis Sequencing, Davis, CA). The pGEM-T-*bb0243* construct was digested with DraII (corresponding to position 248,983 in the *B. burgdorferi* chromosome). A DNA fragment containing *flgB-aadA* (a spectinomycin/streptomycin resistance cassette driven by the *B. burgdorferi flgB* promoter) was amplified from vector pKFSS1 as previously described [64] and inserted into the DraII site within PGEM-T-*bb0243* by blunt-end ligation. The construct was transformed into *E. coli* DH5α and selected by growth on LB agar plates supplemented with 100 µg/ml of spectinomycin. Transformants harboring plasmids containing *bb0243* disrupted by the *flgB-aadA* cassette were isolated, purified to single colonies and plasmid inserts were confirmed by PCR and DNA sequence analysis. *flgB-aadA* cassette orientation was determined by restriction enzyme digestion. A plasmid construct designated pCP100 had the *flgB-aadA* cassette in the same orientation as *bb0243* and a plasmid construct designated pCP200 had the *flgB-aadA* cassette in the reverse orientation. The ampicillin resistance cassette (*bla*) located in pGEM-T was disrupted in both constructs as previously described [65] yielding a plasmid designated pCP101 from pCP100 and pCP201 from pCP200. Spectinomycin-resistant, ampicillin-sensitive colonies of each construct were selected by growth on LB agar containing either 100 µg/ml ampicillin or 100 µg/ml spectinomycin.

**Table 4:** Oligonucleotide primers used in this study.

Name^a^	Sequence (5′ - 3′)	Purpose
243probeF	GATCTTATAATAATTGGAGGGGGC	Southern Hybridization
243probeR	CGCGCAAAGCTTCTTTAAC	
NdeI243F	GGGTTTTCATATGGAGGAATATTTAAATTTCATG	rGlpD generation
XhoI243R	TTTCTCGAGTTAAATTAAATATTTTTTACTTATTTC	
bb0243F	AACAAAGCGGTTGGAAAAGCAAAATCCTG	*bb*0243 disruption
bb0243R	CGATATTTCAGGCTGAAAGTGTCAAAGAGG3	
flgBF	TAATACCCGAGCTTCAAGGAAG	
aadAR	GACGTCATTATTTGCCGACTACC	
bb0240qRTPCRF	AAGTCCCGAAATACCAGGAG	qRT-PCR
bb0240qRTPCRR	TTCTTGCTGCTGTGTAAATACC	
bb0243qRTPCRF	GCTCTGTTCTATATTACGATGATT	qRT-PCR
bb0243qRTPCRR	AGGGCAATGCCTCCTTTTT	
bbb04qRT-PCRF	GGGATTACAGGAGGATTTTTATCTCT	qRT-PCR
bbb04qRT-PCRR	ATTCCCCATTTAGCAGCATCTC	
ospA-288F	TGAAGGCGTAAAAGCTGACAAA	qRT-PCR
ospA-369R	TTCTGTTGATGACTTGTCTTTGGAA	
ospC-B31FTq	CAGGGAAAGATGGGAATACATCTGC	qRT-PCR
ospC-B31RTq	CGCTTCAACCTCTTTCACAGCAAG	
FL-571F	GCAGCTAATGTTGCAAATCTTTTC	qPCR/qRT-PCR
FL-677R	GCAGGTGCTGGCTGTTGA	
flaBFAM	FAM-AAACTGCTCAGGCTGCACCGGTTC-MGB	

a For each primer pair, F refers to forward and R to reverse primer.

pCP101 and pCP201 were isolated and transformed into *B. burgdorferi* B31-A3 competent cells by electroporation as described [66]. Transformants were screened by growth in a 96 well plate in the presence of streptomycin (100 µg/ml). Selected transformants were cloned by limiting dilution in BSK-II medium containing streptomycin (100 µg/ml). The *glpD* disruption in selected transformants was confirmed by Southern blot and Western blot analyses (Figures 8 and 9). Plasmid content for selected mutants was determined as previously described [67] to ensure that all plasmids essential for murine infectivity were present.

### Southern hybridization

Southern blot analysis and generation of a digoxygenin-labeled *bb0243* probe were performed as previously described [67] with the following modifications. A 196 base pair fragment of *bb0243* was generated from strain B31-A3 by PCR using primers 243probeF/R (Table 4). *B. burgdorferi* DNA was fragmented by incubation with 4.5 units of BamHI in 1× buffer B (Fermentas, Glen Burnie, MD) or EcoRI in buffer EcoRI (Fermentas) overnight at 37°C.

### Acquisition of *B. burgdorferi* by *I. scapularis* and transmission to mice

C3H/HeJ mice (Jackson Laboratories, Bar Harbor, ME) were infected with either wild-type or *glpD* mutant *B. burgdorferi* by needle inoculation as previously described [68], [69]. Once infection was established as determined by culture of ear biopsy, mice were anesthetized with ketamine and 100–300 naïve, unfed larvae were placed in and around the ear canal. Mice were placed individually into cages with approximately 1 cm water at the bottom. A metal grid of the same length and width as the cage, and standing 1.5 cm high, was placed in the cage. Larvae were allowed to feed until repletion. Following drop off, larvae were collected, rinsed in water, pooled into groups of 30 in 5 ml tubes with a porous cover and maintained in a desiccator at 21°C, >95% relative humidity with a 16 hour∶8 hour light: dark cycle. Larvae molted to unfed nymphs in approximately 5–6 weeks. At four weeks post molt, three unfed nymphs were placed on three-week old uninfected C3H/HeJ mice (Jackson) and allowed to feed until repletion. Fed nymphs were collected as described above.

For interrupted feeding experiments, 3 unfed nymphs were allowed to feed on naïve C3H/HeJ mice for 24, 48, 55, 62, and 72 hours or to repletion. Ticks were carefully removed from mice by forceps at the indicated time point. Ticks were processed for DNA isolation as described below. Mice were tested for infection as previously described [68], [69].

### DNA isolation

DNA isolation from 5×10^8^ *B. burgdorferi* was performed using the Puregene DNA isolation kit as per manufacturer's instructions (Qiagen, Valencia CA). DNA pellets were resuspended in 30 µl of nuclease free water. DNA concentration was measured by spectrophotometric analysis at 260 nm.

DNA was isolated from pools of 10 fed larvae. DNA was obtained from unfed nymphs that were processed either individually, in groups of 5 or in groups of 10. DNA was isolated from individually processed fed nymphs. Ticks were surface sterilized by washing successively with 800 µl of sterile H_2_O, 0.5% sodium hypochlorite, 3% hydrogen peroxide (Sigma), 70% ethanol (Fischer Scientific, Pittsburgh, PA) and sterile H_2_O each for 1 minute. DNA extraction was performed as adapted from the Qiagen DNeasy blood and tissue kit as described by Beati et al. [70] with the following modifications. Ticks were homogenized with an 18.5 gauge needle. Samples were lysed with 220 µl animal lysis buffer and 0.45 mg recombinant proteinase K (Roche, Mannheim, Germany) per reaction overnight in a 56°C incubator. Following all wash steps, mixtures were centrifuged at 10,000 rpm. DNA was eluted twice with 25 µl of PCR-grade H_2_O pre-warmed to 72°C.

### Quantitative PCR

qPCR reaction mixtures (25 µl total volume) contained 2 µl of sample DNA, 3 µl of nuclease free water, 20 pmol each of primers FL-571F/FL-677R, 5 pmol of *flaB*- specific Taqman probe (flaBFAM) (table 4), and 12.5 µl Taqman PCR mastermix (Roche). DNA copy number was determined on an ABI prism 7900HT thermocycler with an amplification profile of 50°C for 2 minutes, 95°C for 10 minutes, followed by 40 cycles of 95°C for 15 seconds and 60°C for 1 minute. Samples were run in duplicate and each plate contained two samples lacking DNA as negative controls. Ct values were obtained using the SDS2.1 software program (Applied Biosystems, Carlsbad, CA). To assess spirochete density per sample, standard curves were generated for *flaB*, a constitutively expressed gene, in log increments (10–10^4^).

Copy numbers were compared by a two-tailed, unpaired *t*-test for each condition (fed larvae, unfed nymph, fed nymph), where significance was defined as *P*≤0.05.

### RNA extraction

Ticks infected with *B. burgdorferi* were processed in pools of 50 for fed larvae, 100 for unfed nymphs and 35 for fed nymphs. Ticks were homogenized in 1 ml TRIzol reagent (Invitrogen, Carlsbad, CA) for 5 minutes. For in vitro experiments, 50 ml of cell culture (approximately 2.5×10^9^ cells) was centrifuged at 12,000 rpm for 10 minutes and 1 ml TRIzol was added to the cell pellet. RNA was recovered as per manufacturer's instructions (Invitrogen). The RNA pellet was resuspended in 30 µl nuclease-free water and DNase treated twice with the Ambion DNA free kit per manufacturer's instructions (Ambion, Austin, TX).

Mammalian hind limb joints were surgically removed from euthanized C3H/HeJ mice, snap frozen in liquid nitrogen and pulverized with mortar and pestle. The powdered tissue was transferred to a glass homogenizer and homogenized with 0.5 ml denaturation solution and the supernatant containing total RNA was isolated from samples as per manufacturer's instructions (ToTALLY RNA, Ambion). RNA was rehydrated in 30 µl of nuclease free water and DNase treated as described above. Mouse RNA was removed by MICROBE*nrich* as per manufacturer's instructions (Ambion). The recovered RNA pellet was resuspended in 15 µl of sodium citrate buffer (Ambion) and 15 µl of nuclease free water.

cDNA was generated from RNA samples by addition of 2 µg of purified RNA to a mixture containing 4 µl of 5× reverse transcriptase buffer (Promega), 0.02 mM dNTPs (Roche), 0.5 µg random hexamer (Promega), 2 units of RNase inhibitor (Ambion), 5 units of AMV reverse transcriptase enzyme (Promega) and nuclease free water in 20 µl total volume. The reaction mixture was incubated at 42°C for 2 hours. Reverse transcriptase enzyme was heat inactivated at 95°C for 5 minutes and cDNA was stored at −20°C until further use.

### Quantitative RT-PCR

For generation of standard curves, specific gene fragments for *bb0240*, *bb0243* and *bbb04* were amplified by PCR using primers pairs bb0240qRTPCRF/bb0240qRTPCRR, bb0243qRTPCRF/bb0243qRTPCRR, bbb04qRT-PCRF/bbb04qRT-PCRR, ospA-288F/ospA-369R and ospC-B31FTq/ospC-B31RTq, respectively (table 4). PCR reaction mixtures contained 100 ng of B31-A3 DNA, 0.25 µl Taq polymerase (Roche), 0.5 µl dNTPs (Roche), and 1× Taq polymerase buffer (Roche) in a total volume of 25 µl. Amplification conditions were 95°C for 5 minutes, followed by 36 cycles of 95°C for 30 seconds, 55°C for 30 seconds and 72°C for 30 seconds and a final incubation at 72°C for 10 minutes. Production of the expected product was confirmed by gel electrophoresis and the PCR products were ligated into the TOPO 2.1 cloning vector, the vector was transformed into *E. coli* Mach1 cells and recombinant clones were selected as per manufacturer's instructions (Invitrogen). Clonal isolates were grown in 10 ml of LB broth supplemented with 100 µg/ml ampicillin and the plasmids were extracted as described above. PCR confirmed the presence of the desired gene fragments. Plasmid concentration was determined by spectrophotometric analysis at 260 nm, followed by mathematical computation of copy number (http://www.uri.edu/research/gsc/resources/cndna.html).

Transcript levels for *bb0240 (glpF)*, *bb0243* (*glpD*), *bbb04* (*chbC*), *ospA*, *ospC* and *flaB* were determined by performing qRT-PCR as previously described [65] using the primer pairs listed in table 4 on an ABI Prism 7900HT thermocycler followed by analysis using the SDS2.1 software program (Applied Biosystems). For each experimental run, standard curves for these genes were generated using known quantities (10–10^4^ in log increments) of gene specific plasmids for calculation of absolute copy number.

One-way analysis of variance was performed on qRT-PCR results. To determine significance, a Kruskal-Wallis multiple comparison Z-value test (Dunn's test) was performed, where significance was defined as *P*≤0.05.

### Production of recombinant GlpD (rGlpD) and GlpD antibodies

*bb0243* was amplified using primers NdeI243F/XhoI243R (Table 4), cloned into the TOPO 2.1 vector, transformed into *E. coli* Top10 cells and recombinant clones were selected per manufacturer's instructions (Invitrogen). The *bb0243* insert was excised from the TOPO 2.1 plasmid by double digestion with NdeI and XhoI (Fermentas) and the insert was purified after separation by gel electrophoresis using the Wizard SV genomic gel purification kit according to manufacturer's instructions (Promega). pET-15b (Novagen, Gibbstown, NJ) was digested with NdeI and XhoI and the gel-purified *bb0243* insert was ligated with the NdeI/XhoI-cut pET-15b at a 2∶1 ratio with 10 units of T4 ligase (New England Biolabs, Ipswich, MA). The recombinant plasmid was transformed into *E. coli* DH5α and clones were selected on LB agar plates containing 100 µg/ml ampicillin. Recombinant pET-15b carrying *bb0243* was transformed into *E. coli* BL21-DE3 and grown on LB agar plates containing 100 µg/ml ampicillin. A clone containing *bb0243* was selected and subjected to DNA sequencing. This sequence contained two single nucleotide changes relative to the reported sequence in strain B31-MI [12]. Nucleotide 107 had a T to C change that would result in a predicted amino acid change of I359T and nucleotide 591 had an A to T substitution that would result in an amino acid change of E197D.

The selected clone was grown at 37°C in 250 ml Luria broth containing 100 µg/ml ampicillin and 1 mM IPTG with agitation for 4 hours. Cells were recovered by centrifugation at 8000 RPM for 10 minutes and rGlpD was isolated from the cells using Ni-NTA His Bind Resin (Novagen) according to the manufacturer's instructions. Fractions containing rGlpD, as determined by SDS-PAGE, were pooled and loaded into an Amicon Ultra 50 kDa molecular weight cut off spin column (Millipore, Billerica, MA). The protein sample was centrifuged at 7500× g for approximately 8 minutes to a volume of 800 µl. The protein solution was dialyzed against 2 liters of 1× PBS, 6 M urea (pH 7.4) with stirring overnight at 4°C. Identity of the protein as *B. burgdorferi* rGlpD was confirmed by LC-MS/MS analysis (Keck Biotechnology Resource Laboratory,New Haven, CT). The yield of purified rGlpD was 1.3 mg.

100 µg of purified rGlpD in 1×PBS, 6 M urea (pH 7.4) was inoculated along with Freund's adjuvant into two Sprague-Dawley rats by Harlan Laboratories (Madison, WI). The rats received a boost at day 28 and day 56 post-inoculation and were sacrificed and bled on day 70 post inoculation. GlpD antiserum was tested by ELISA and confirmed to be specific for GlpD by immunoblot analysis.

### SDS-PAGE and immunoblot analysis

*B. burgdorferi* cells grown in vitro or in DMCs were lysed with Bugbuster HT (Novagen) and 1 µg/ml of lysozyme (Sigma) according to manufacturer's instructions. 2 µg of whole cell lysate was subjected to 12.5% SDS-PAGE and separated proteins were visualized by silver staining as described [71]. For immunoblotting, separated proteins were transferred to PVDF membrane. Membranes were exposed to protein-specific primary rat antiserum (GlpD, 1∶400 dilution; FlaB, 1∶2500 dilution) followed by alkaline phosphatase-linked anti-rat secondary antibody (1∶500) (KPL, Gaithersburg, MD). The membrane was washed three times for 10 minutes with 1× TBS/0.05% Tween 20 and developed with BCIP/NBT phosphatase substrate (KPL) until band development (approximately 2–4 minutes).

To determine seroconversion in mouse infection studies, mouse serum was added to 1× TBS with 0.5% dry milk at 1∶200 dilution and incubated with whole *B. burgdorferi* lysate Marblot strips (MarDX, Jamestown, NY) for 1 hour at room temperature. The remaining procedure is as described above, with anti-mouse secondary antibody (1∶5000 dilution).
